# Recent Advances in the Use of *Drosophila melanogaster* as a Model to Study Immunopathogenesis of Medically Important Filamentous Fungi

**DOI:** 10.1155/2012/583792

**Published:** 2012-02-19

**Authors:** Georgios Hamilos, George Samonis, Dimitrios P. Kontoyiannis

**Affiliations:** ^1^Department of Internal Medicine, School of Medicine, University of Crete, Stavrakia, Voutes, 71110 Heraklion, Crete, Greece; ^2^Department of Infectious Diseases, Infection Control and Employee Health, The University of Texas MD Anderson Cancer Center, 1515 Holcombe Boulevard, Houston, TX 77030, USA

## Abstract

Airborne opportunistic fungi, including *Aspergillus* and other less common saprophytic molds, have recently emerged as important causes of mortality in immunocompromised individuals. Understanding the molecular mechanisms of host-fungal interplay in robust experimental pathosystems is becoming a research priority for development of novel therapeutics to combat these devastating infections. Over the past decade, invertebrate hosts with evolutionarily conserved innate immune signaling pathways and powerful genetics, such as *Drosophila melanogaster*, have been employed as a means to overcome logistic restrains associated with the use mammalian models of fungal infections. Recent studies in *Drosophila* models of filamentous fungi demonstrated that several genes implicated in fungal virulence in mammals also play a similarly important pathogenic role in fruit flies, and important host-related aspects in fungal pathogenesis are evolutionarily conserved. In view of recent advances in *Drosophila* genetics, fruit flies will become an invaluable surrogate model to study immunopathogenesis of fungal diseases.

## 1. Introduction

In recent years, opportunistic fungi have emerged as leading causes of morbidity and mortality in immunocompromised individuals [1–3]. *Aspergillus* is by far the most common of these molds, and mortality rates for invasive aspergillosis exceed 90% in hematopoietic stem cell transplant recipients [4, 5]. Even more concerning, however, is that infections caused by other difficult-to-treat opportunistic molds, such as Mucorales species, are increasingly being observed in several cancer centers [6–8]. The increase in the frequency and spectrum of invasive fungal infections in immunocompromised patients underscores the need for expanding our knowledge of the pathogenesis of opportunistic fungal infections and developing novel therapeutic approaches.

The versatility and complexity of virulence mechanisms and predisposing host conditions that lead to development of invasive mold infections [9, 10] necessitate understanding the nature of host-fungal interactions at the cellular and molecular levels in order to identify host immune pathways and pathogen determinants involved in disease progression [11, 12]. Pioneering studies over the past decade demonstrated that a variety of opportunistic fungi can invade and cause fatal infection in a variety of simple invertebrate hosts, such as the fruit fly *Drosophila melanogaster*, and the roundworm *Caenorhabditis elegans* [13–20]. Also, it has become evident from these studies that important aspects of innate immunity have been evolutionarily conserved across phylogeny. Thus, because of their simplicity, well characterized innate immune signaling pathways, and because both the host and pathogen are amenable to genetic analysis and high-throughput screening in each of these pathosystems, the use of invertebrate models has accelerated studies of microbial virulence and host immunity [21–24]. In addition, because of their low cost, small size, and short generation time, invertebrate hosts have been used in mass screening assays for selection of antimicrobial compounds with novel mechanisms of action. In this review, we outline recent advances in the study of medically important filamentous fungi in *Drosophila* model and discuss future implications and challenges in the use of this elegant pathosystem.

## 2. Antifungal Innate Immune Pathways in *Drosophila melanogaster*

### 2.1. Humoral Antifungal Immune Responses

Although lacking adaptive immunity, invertebrates are capable of having efficient innate immune responses against an array of pathogens in their natural environments. Two major pathways orchestrate innate immune responses in *D. melanogaster*, the immune deficiency (*imd*) pathway that confers protection against gram-negative bacteria, and the *Toll* pathway that is critical for immunity against gram-positive bacteria and fungi [25]. Detection of invading microorganisms by host receptors of the peptidoglycan recognition protein (PGRP) or gram-negative binding protein (GNBP) families triggers the activation of signal transduction pathways in the fat body (liver analogue) via the *Toll* receptor leading to a systemic humoral response characterized primarily by massive synthesis and release of potent antimicrobial peptides. Despite the broad spectrum of antimicrobial peptides, some specificity exists upon their induction following infection by various microbial pathogens. For example, in *D. melanogaster*, fungi and gram-positive bacteria mainly induce the production and release of drosomycin and metchnikowin via the *Toll *pathway, whereas gram-negative microbes induce the production and release of diptericin, attacin, and cecropin via the *imd *pathway [25]. The predominant role of the *Toll* pathway in *Drosophila* immunity against *Aspergillus* was first demonstrated in a landmark study by Lemaitre et al. [17], who found that *Toll* mutant flies, in contrast to wild-type flies, were highly susceptible to *Aspergillus* infection.

In both insects and mammals, the interaction of immunostimulatory cell wall molecules of invading fungi with *Toll* receptor(s) leads to activation of intracellular phosphorylation cascades, with subsequent translocation of nuclear factor *κ*B-like transcriptional factors to the nucleus and induction of antimicrobial peptide-encoding genes [10, 25]. However, in contrast to mammalian *Toll* receptors, there is no direct interaction between microbial ligands and *Drosophila Toll* receptor. Instead, activation of the *Toll* signaling cascade is mediated by GNBP-3, a soluble pattern recognition receptor that senses long-chain fungal b-(1–3)-glucans and triggers a serine protease cascade leading to the processing of a small cytokine-like molecule, *Spätzle*, which comprises the functional ligand of the *Toll* receptor [25]. Importantly, GNBP3 mutant flies are highly susceptible to opportunistic fungi, including *Candida* and *Aspergillus* while retain functional *Toll* pathway activity, implying *Toll*-independent immune-related functions of this receptor. Indeed, GNBP3 has been implicated to play a role in pathogen agglutination, and activation of the melanization reaction at the early stages of fungal invasion [26]. Importantly, parallel to GNBP3, a second detection system senses the activity of proteolytic virulence factors that are released in the fly hemolymph during invasive fungal growth and redundantly activates *Toll* pathway via the protease *Persephone *[27].

### 2.2. Cellular Antifungal Immune Responses

When compared to humoral immune responses,* D. melanogaster* cellular immune responses are less well characterized. Notably, recent studies in insects challenge the importance of humoral immunity in pathogen clearance, demonstrating that the vast majority of bacteria (99.5%) are rapidly eliminated from the haemolymph well before the induction of antimicrobial peptides [28]. Hence, cellular immune responses seem to play instrumental roles in early recognition and elimination of microbial pathogens. The key transcription factor downstream of the *Toll *pathway, the nuclear factor-*κ*B homologue Dif, is required for regulation of both humoral and cellular immunity in flies [25]. Phagocytosis is a hallmark of the cellular immune response and exhibits considerable similarity across phylogeny. Hence, opsonization and recognition by specific receptors mediate the initial stages of phagocytosis in both invertebrates and mammals. For example, in *Drosophila* peptidoglycan, recognition proteins (PGRPs) such as PGRP-LC and Croquemort (a human CD36 homologue) participate in the recognition and phagocytosis of gram-negative bacteria [29, 30], whereas the transmembrane scavenger receptor *eater* has been shown to recognize bacteria and fungi (*Candida silvata*) and play a *Toll*-independent role in antifungal immunity [23, 31]. Of interest, thioester-containing proteins with a complement-like activity against invading pathogens have been identified in many insects, including fruit flies [25, 32]. A high-throughput screen in *Drosophila melanogaster* S2 RNAi library identified a novel protein, macroglobulin complement related (*Mcr*), that exerts opsonizing activity specifically against *Candida albicans *[33]. In addition, S2 *Drosophila* cells efficiently eliminate *C. albicans* yeast cells and induce significant damage to the hyphae of filamentous fungi, including *Aspergillus* and the *Mucorales*, in a way that resembles the antifungal effector function of human phagocytes [23, 34].

The molecular mechanisms of intracellular elimination of pathogens by *Drosophila* phagocytic cells are less well characterized. Thus, insect phagocytic cells are also capable of generating an oxidative burst of oxygen radical intermediates, whereas induction of nitric oxide synthase has been shown to protect against bacterial infection in *Drosophila* larvae [35]. Furthermore, numerous antimicrobial peptides contained within human neutrophil granules, such as lysozyme, lipases, metalloproteases (like the mammalian gelatinases or collagenases), and nucleases, are similarly produced by the phagocytic hemocytes of most insects in response to infection [11, 25]. Although little is known about the molecular mechanisms of intracellular elimination of pathogens in fruit flies, recent studies demonstrate that the evolutionarily conserved autophagy pathway is important for immune surveillance and clearance of intracellular pathogens that escape into the cytoplasm, including *Cryptococcus* [36]. On the other hand, unique cellular responses against larger invading pathogens (e.g., parasites), such as encapsulation and melanization mediated by specialized immune effector cells, are seen in *Drosophila* and other insects [11, 25].

### 2.3. Epithelial Immune Responses

In *D. melanogaster*, antimicrobial peptide-encoding genes are constitutively expressed in epithelia that are in direct contact with the external environment. However, in contrast with the systemic immune responses mediated by the fat body, where the *Toll *pathway modulates immune responses against gram-positive bacteria and fungi, epithelial immune responses in *D. melanogaster *appear to be partially controlled by the *Imd *pathway [25]. Furthermore, recent evidence indicates that genes involved in oxidative stress and/or detoxification of reactive oxygen species are critical for epithelial defense [37]. In addition, recent studies demonstrated a major role for the Janus kinase- (JAK-) signal transducer and activator of transcription (STAT) signaling pathway in epithelial host defense via regulation of stem cell proliferation and epithelial cell homeostasis [38].

A recent study in a gastrointestinal infection (GI) model of candidiasis in *Drosophila* larvae demonstrated an important role of normal gut flora in epithelial immunity by preventing colonization and invasive infection by *Candida, *which resembles the increasingly appreciated regulatory role of human epithelial microbiota in shaping epithelial immune responses [39]. Of interest, activation of JNK signaling during *Candida *infection accounted for extensive epithelial cell death in the gut and mortality of *Drosophila* larvae. In parallel, *Candida* infection triggered a systemic protective immune response that was mediated by NO release from larvae hemocytes and the parallel activation of the *Toll* pathway by pathogen-secreted aspartyl proteinases.

### 2.4. Toll-Independent Innate Immune Pathways in *Drosophila*

The complexity of the immune defenses in insects is much higher than initially perceived, and cross-talk between the *Imd *and *Toll *pathways takes place in response to both gram-negative and gram-positive microbes [25]. Furthermore, besides the *Toll *and *Imd *signaling cascades, other pathways associated with developmental or stress resistance processes are induced in response to infections in both invertebrates and mammals. For example, a pioneer study in *Drosophila *demonstrated that antimicrobial peptide activation can be achieved independently of classic immunoregulatory pathways by the transcription factor FOXO, a key regulator of stress resistance, metabolism, and aging [40]. In uninfected animals, antimicrobial peptide genes are activated in response to nuclear FOXO activity when induced by starvation or by using insulin-signaling mutants, revealing a new mechanism of cross-regulation of metabolism and innate immunity that has proven to be functional in humans as well [40]. Furthermore, investigators showed that the activation of the evolutionarily conserved p38 MAPK pathway is important for resistance to infection by bacteria and fungi; of interest, in contrast to the mammalian homologue, activation of p38 MAPK occurs independently of the *Toll* signaling [41].

## 3. Modeling Microbial Infection in *Drosophila melanogaster*

In *D*. *melanogaster,* the pathogen of interest is typically injected into the dorsal thorax via either needle pricking or microinjection [11]. In regard to fungal pathogens, the injection assay is technically a more standardized and reproducible method of infection and allows for a more precise estimation of fungal inoculums. Nonetheless, parenteral inoculation by passes the physiologic route of entry of the pathogen of interest and results in a more overwhelming infection that may not be suitable for pathogenesis studies. Thus, other more physiologic methods of infection are also used. For example, the *alb1 Aspergillus fumigatus* mutant, which is hypovirulent in mice, exhibited attenuated virulence in *Toll*-deficient flies only when introduced by feeding or rolling [20]. These infection methods are typically achieved by feeding insects in a lawn of yeast or molds or rolling insects over a fresh carpet of fungal spores. However, standardization of the infecting inocula is difficult with natural infection methods such as ingestion. Furthermore, infection with molds other than *Aspergillus by *feeding and rolling is difficult to perform because of the distinct pattern of growth of fungal colonies.

Female flies are typically used in infection experiments because of their larger size and relative resistance to injection injury when compared with male flies. Because wild-type *Drosophila* is resistant to most pathogenic fungi and bacteria, mutants deficient in various components of the *Toll* cascade are frequently employed to model infections. In most cases, crossing different loss-of-function alleles is required to generate homozygous *Toll*-mutant flies [11]. Nonetheless, the need for crossing of fly strains is a limitation for high throughput screening assays. Of note, microinjection introduces significantly higher inoculums within *Drosophila *hemolymph than needle pricking that allowed for establishment of invasive *Candida* infection in wild-type *Drosophila melanogaster* flies [42].

A major advantage of *Drosophila* in comparison to all other model host organisms is its genetic tractability, well-characterized immune system, and remarkable degree in conservation of biochemical pathways that control fundamental physiologic processes such as cell proliferation, differentiation, and tissue homeostasis. Furthermore, the innate susceptibility of *Drosophila Toll *mutant strains to fungal infections obviates the need to use immunosuppressive agents, thus eliminating the host variability inherent in the use of immunosuppressive regimens. In particular, *Drosophila* strains are amenable to both forward and reverse genetics, and large collections of *Drosophila *mutants and transgenic cell lines are commercially available (http://flybase.net/). Also, the *Drosophila* genome sequence was one of the first to be completed and is probably one of the most fully annotated eukaryotic genomes found in a database (http://flybase.net/annot/). As a result, double-stranded RNA has been synthesized for each of the *Drosophila* genes (http://www.flyrnai.org/) and recently lines expressing RNAi have become available, which allow for conditional inactivation of every single gene at a whole animal or tissue level (http://www.vdrc.at/).

## 4. Virulence Studies of *Filamentous Fungi *in *Drosophila melanogaster*

### 4.1. Aspergillus

Since filamentous fungi have been in existence for about 1 billion years, the fly immune system evolved in the face of continued exposure to airborne conidia. Thus, *Drosophila* immune system has developed highly sophisticated and efficient strategies to combat infection caused by *Aspergillus* and other filamentous fungi. In fact, only a few entomopathogenic fungi are able to infect fruit flies in nature, via penetration of fly exoskeleton. Even when fungal pathogens are experimentally introduced directly into the fly hemolymph, wild-type flies are still capable of effectively eliminating infection. Lemaitre and colleagues were the first to demonstrate that *Aspergillus fumigatus* was able to infect and kill flies carrying mutations in various aspects of the *Toll* pathway [17]. *Toll*-deficient flies have been since implemented as a model to study immunopathogenesis of infections caused by *Aspergillus* and other medically important filamentous fungi. Several virulence attributes of *Aspergillus* pathogenicity in mammals have been tested in the fly model [20, 43]. With the exception of virulent factors that are important for microbial survival at mammalian temperature [44], most other virulence attributes that are important for mammalian pathogenicity of *Aspergillus* were equally important for successful infection in *Toll*-deficient fruit flies. In particular, *Aspergillus* mutants that are defective in siderophore biosynthesis (DeltasidA, DeltasidD), PABA metabolism (H515), starvation stress response, secondary metabolite production (DgliP), or melanin biosynthesis were attenuated in both *Drosophila and mouse* models of invasive aspergillosis [20, 43]. Notably, fungal cell wall melanin was dispensable for *Aspergillus* virulence when fungal spores were injected into the fly hemolymph but was important for establishment of invasive infection though *Drosophila* epithelia [20]. Hence, the tempo and site of infection as well as differences in local host defense mechanisms may influence expression of virulence factors of fungi in the fly model. Evermore, similar to recent findings with the Δ *CgrA *mutant [44], putative virulent factors of *A. fumigatus* with a role in thermotolerance may not be encountered in *Drosophila* or other invertebrate models because infection in these minihosts takes place at temperatures much lower (25°C) than the mammalian physiologic temperature (37°C). Despite these limitations, accumulating experimental evidence suggests that *Drosophila* is a relevance model to study *Aspergillus* virulence.

The interstrain and interspecies variations in virulence for a collection of *Aspergillus fumigatus *and *Aspergillus terreus* clinical isolates were recently studied in *Toll*-deficient fruit flies [45]. Although there was no significant difference in the survival of flies infected with *A. fumigatus* versus* A. terreus* or flies infected with colonizing versus invasive isolates of either species, two dominant A. fumigatus clades identified by rep-PCR were associated with significantly different survival rates in *Toll*-deficient flies. Therefore, the fly model of aspergillosis could detect subtle changes in virulence and uncover distinct *A. fumigatus* clades that differ in their pathogenicity. Of interest, a similar pathogenicity study of *Candida albicans* clinical isolates that were previously ranked for virulence in mice was recently performed in wild-type *Drosophila* flies infected by microinjection [42]. Of interest, there was a significant correlation in virulence of *C. albicans* strains between the fly and the mouse model of disseminated candidiasis. Nonetheless, differences in virulence were not evident using immune-deficient spatzle^-/-  ^flies, suggesting that *Toll* signalling might actually be required to predictably differentiate virulence.

The recent completion of the sequencing of the *A. fumigatus* genome and the development of molecular toolsets to study the biology of *A. fumigatus* is expected to lead to the generation of multiple *Aspergillus* mutants and creates a need for high-throughput strategies capable of assessing the contribution of individual genes to *Aspergillus* virulence [46]. Validation of *Drosophila* as a suitable model for large-scale virulence studies was provided by a recent screen of 34 *Candida albicans* mutants defective in putative transcription factor genes. This study identified a novel transcriptional regulator of cell wall integrity, CAS5, which proved to be important for virulence in both *Drosophila* and the mouse model of invasive candidiasis; a parallel screen in *C. elegans* subsequently confirmed the role of CAS5 in *Candida* virulence [47].

### 4.2. Mucorales (Formerly Zygomycetes)

Mucorales species have recently emerged as an important cause of serious angioinvasive infections in immunocompromised individuals [6–8]. *Rhizopus* species accounts for majority of cases of mucormycosis in humans [7]. Few animal models of mucormycosis exist, and the immunopathogenesis of this infection is largely unknown. However, sequencing of *Rhizopus oryzae* genome has been completed and genetic tools are available (http://www.broad.mit.edu/annotation/fungi/rhizopus_oryzae/). In immunocompetent individuals, blood and tissue phagocytes efficiently eliminate *Mucorales* spores and hyphae by oxidative and nonoxidative killing mechanisms. Quantitative (i.e., neutropenia) or qualitative (i.e., associated with glucocorticoids, hyperglycemia, and/or acidosis) defects in phagocytic cell activity permit unrestricted growth of the hyphal form and invasive infection. Iron metabolism has a central role in pathogenesis of mucormycosis [6–8]. Thus, patients with iron overload states, including individuals undergoing chelation therapy with deferoxamine, are uniquely predisposed to mucormycosis. Of interest, deferoxamine acts as a siderophore for *Mucorales *species and promotes *in vitro* fungal growth. Similarly, the increased availability of serum iron in patients with diabetic acidosis partially accounts for their unique susceptibility to mucormycosis. As opposite to deferoxamine, other iron chelators such as deferasirox lack xenosiderophore activity for *Rhizopus* induce an iron-starvation effect to the fungus and have shown to be protective in animal models of mucormycosis [6–8].

Although Mucorales have not be reported to be entomopathogenic, we recently found that as opposite to other medically important filamentous fungi, injection of different Mucorales species in wild-type *D. melanogaster* results in a hyperacute infection, with disseminated fungal proliferation and high mortality rates [23]. Several aspects of immunopathogenesis of mucormycosis in humans were modeled in *Drosophila*, including increased host susceptibility following administration of corticosteroids, and the iron chelator deferoxamine. Of interest, the use of another iron chelator, deferasirox, which induces iron starvation to Mucorales spp and protects mice and possibly humans from infection, also significantly protected *Drosophila* from mucormycosis. In addition, *Cunninghamella berthollethiae*, which appears to be the most virulent Mucorales species in humans, exhibited increased virulence in comparison to other Mucorales species in the fly model [23].

The fly model of mucormycosis has been established in wild-type *Drosophila*, which obviates the need for crossings and allows for simple and rapid assessment of research questions in *Mucorales* pathogenicity. Thus, flies were recently implemented to evaluate the role of endosymbiotic toxin-producing bacteria in the virulence of *Rhizopus *species. Although a significant number of clinical *Rhizopus* isolates were found to harbor rhizoxin-producing bacteria, there was no difference in fungal virulence following antibiotic mediated eradication of the endosimbionts in both *Drosophila* and mice [48]. In addition, the association of increased voriconazole use with the emergence of Mucorales infection in immunocompromised patients was recently tested in the fly model. Surprisingly, preexposure of Mucorales to this newer triazole dramatically increased susceptibility of fruit flies to mucormycosis in *Toll*-independent fashion, which was also observed in the mouse model [49]. Collectively, these studies demonstrate that Mucorales species have developed common virulence strategies to invade evolutionarily disparate organisms such as *Drosophila* and humans.

Of interest, virulence of *Cunninghamella* in the fly model is significantly affected by the composition of fungal culture media, possibly reflecting differences in acquisition of iron or other nutritional factors [50]. In addition, because innate immunity in *Drosophila* is under circadian regulation, the timing of infection has significant impact in host defense against various pathogens, including filamentous fungi. In fact, genes involved in circadian rhythm regulation were significantly induced following infection with Mucorales species in *Drosophila* [23]. Furthermore, starvation of flies prior to infection confers protection against bacterial infection via release of NO [51], and possibly via regulating other immune-related pathways, such as FOXO signaling [40] and the autophagy response. Therefore, all these parameters need to be considered in virulence testing of *Mucorales* and other filamentous fungi in *Drosophila*.

Gene expression profiling in human monocytes and in immunocompromised mice infected with *Rhizopus* versus *Aspergillus* demonstrates a differential induction of immune-related genes during mucormycosis [52], which likely reflects unique virulence traits of Mucorales species. Similarly, transcriptional profiling at early time points of infection in wild-type fruit flies infected with *Rhizopus *(pathogenic) versus *Aspergillus *(nonpathogenic) indicated distinct sets of genes that were selectively regulated in response to mucormycosis [23]. These genes could represent molecular targets for drug development aiming at modulating host immune response during infection. Of interest, a similar transcriptome profiling in *Drosophila* infected with two strains of *Pseudomonas* with different pathogenic properties revealed common groups of genes with those identified during *Rhizopus* infection of flies [53]. Notably, a group of genes down regulated following infection with the pathogenic strain in both studies included a skeletal muscle gene regulatory network under the control of cJun-N-terminal Kinase (JNK) pathway. Notably, activation of this pathway promoted local resistance to *P. aeruginosa* in flies and mice [54].

### 4.3. Other Emerging Filamentous Fungi

*Fusarium *and* Scedosporium* species are ubiquitous, saprophytic molds that are notoriously resistant to conventional antifungal agents [2]. These fungi have been increasingly reported causes of invasive, frequently fatal infections in immunosuppressed hosts. Occasionally, these opportunistic pathogens can cause difficult-to-treat localized infections in immunocompetent individuals with certain predisposing conditions, including onychomycosis, fungal keratitis, skin and soft tissue infection, and rarely brain abscesses [2]. Furthermore, as opposite to other filamentous fungi, *Fusarium* species have a unique predisposition for development of fungemia and disseminated necrotic skin lesions in severely immunocompromised patients [2]. These features suggest the existence of uncharacterized, unique virulence factors of these organisms. *Drosophila melanogaster* wild-type flies were recently found to be resistant to infection by different clinical isolates of *Scedosporium*, whereas *Toll*-deficient flies were highly susceptible to these fungi [24]. Of interest, *Fusarium* species caused lethal infection in wild-type flies although in a less acute mode of infection than in *Toll* deficient flies, an observation consistent with the ability of these fungi to infect a broad range of phylogenetically disparate hosts, ranging from plants to mammals. Although the lack of genetic tools currently precludes comprehensive analysis of virulence factors in these fungi, comparative analysis of host defense mechanisms during infection with these and other filamentous fungi in the *Drosophila *model could provide valuable information on the pathogenesis of these emerging infections.

## 5. Antifungal Drug Efficacy Studies in *Drosophila* Models of Filamentous Fungi

*Drosophila* has proven to be a reliable model for testing orally absorbed compounds with antifungal activity. In particular, voriconazole conferred significant protection in* Toll*-mutant flies infected with *A. fumigatus* [20]. Furthermore, the combination of voriconazole and terbinafine, two drugs that block sequential steps in the ergosterol pathway and show synergy *in vitro* against *Aspergillus*, was synergistic in the *Drosophila *model of aspergillosis [20]. Similarly, voriconazole preexposure was protective in flies infected with *Fusarium moniliforme* and *S. apiospermum, *but not in flies infected with *S. prolificans*, a finding that is consistent with *in vitro* susceptibilities of these species and *in vivo* studies in mice [24]. Besides conventional antifungal agents, administration of deferasirox, an iron chelator that induces iron starvation and exerts selective antifungal activity against Mucorales, significantly increased survival of flies in *Drosophila* model of mucormycosis [23].

Nonetheless, there are important limitations in the use of *Drosophila* and other invertebrate models in drug efficacy studies. Thus, precise estimation of the dose of a pharmacologic compound that is orally administered in flies is challenging. A more accurate way of drug delivery can be achieved by microinjection; however, this method is time consuming and requires technical training and specialized equipment in fruit flies. In addition, measurement of drug levels for pharmacokinetic analysis in *Drosophila* requires HPLC or bioassay methods that are more cumbersome, imprecise, and technically demanding in this model than in mammals [11]. For all these reasons, pharmacodynamic studies, which typically require multiple dosing of antifungal agents for long periods of time, are not feasible in *Drosophila*. Finally, the metabolism and elimination pathways of drugs and the potential for drug-drug interactions are largely unknown in *Drosophila *for most existing compounds.

Despite their limitations, *Drosophila* and other invertebrates are attractive models for mass-screening candidate antifungal compounds that will require subsequent validation in mammalian systems [55]. Such approaches have been used successfully in *Drosophila* to select life-extending compounds [56] and recently in *C. elegans* to identify compounds with novel mechanism of antifungal activity against *Candida* [57]. In the *C. elegans* study, thousands of synthetic and natural molecules were screened in a 96-well plate liquid culture system and several compounds that exhibited *in vivo* activity without significant *in vitro* effect were selected, proving the benefits of this strategy. Notably, two of the 15 selected compounds identified in this screening exhibited potent antifungal activity in the mouse model of invasive candidiasis [57]. Overall, the simplicity, low cost, small size, and short generation time of invertebrate hosts make them ideal for high-throughput screening. As a proof of principle, many pharmaceutical and biotechnology companies are increasingly using minihost models for drug discovery. For example, Exelixis, Inc. (South San Francisco, CA) has created an extensive collection of *Drosophila *gene disruption strains for use in drug-target identification. Similarly, larger pharmaceutical companies such as Novartis (Basel, Switzerland) have created *Drosophila *functional genomics departments dedicated to the study of disease-related pathways and discovery of novel drug targets. Nonetheless, *D. melanogaster *models of infectious diseases are less amenable to automated mass screening for antimicrobial agents than are *C. elegans *models because of technical limitations associated with the size of the animals, methods of infection, frequent need for fly crosses to generate the desired mutants, and inability of adult flies to propagate in liquid culture systems.

## 6. Implementing RNAi Screens to Identify Host and Pathogen Determinants of Immunopathogenesis of Fungal Diseases

Over the past few years*, Drosophila melanogaster* S2 cells and RNAi technology have been successfully implemented to identify host factors implicated in pathogenesis of infections caused by intracellular pathogens [29, 30, 58]. There are many features of the *Drosophila* cell system that make it an attractive tool for these studies. Hence, the fly genome is highly annotated and fundamental innate immune pathways are evolutionarily conserved in* Drosophila* S2 macrophage-like cells. Furthermore, gene silencing is easier to perform in a high-throughput basis in *Drosophila* cell lines when compared to mammalian macrophage cell lines. Finally, *Drosophila* S2 cells have a successful track record in identifying novel host factors involved in phagocytosis and killing of many intracellular microbial pathogens, which have been subsequently validated in their mammalian cell counterparts [29, 30, 58]. In regard to fungal pathogens, investigators recently used an RNAi library of S2 cells to study genes involved in phagocytosis of *C. albicans *and identified novel genes encoding for proteins that specifically recognize and promote phagocytosis of *Candida* yeast cells [33]. Another RNAi screen in S2 cells was designed to select host factors that restrict intracellular survival and proliferation of the pathogenic fungus*, Cryptococcus neoformans* [36]. This study identified novel host genes implicated in *Cryptococcus* pathogenesis and revealed that proteins of the autophagy pathway are important for intracellular elimination of the fungus both in *Drosophila* S2 cells and mammalian macrophages.

*In vitro, *high-throughput screening strategies using phagocytic *D. melanogaster *cell lines have certain limitations. First, only host factors important for the intracellular life cycle of a pathogen can be tested. This approach is well suited for intracellular pathogens but not for extracellular organisms such as filamentous fungi. Thus, in contrast with bacteria, fungi have distinct replication stages (e.g., spore to hyphal transition) and relatively slow growth rates, which make difficult the establishment of reliable high-throughput phagocytosis and/or killing *in vitro *assays. In addition, silencing of important innate immune-related pathways may be missed in an *in vitro *screen because it may result in nonviable phenotypes, which can only be assessed using tissue-specific silencing *in vivo*. Finally, the complexity and dynamics of *in vivo* host-pathogen interplay, including tissue-specific host immune responses, cannot be reliably evaluated using an *in vitro* culture system.

Studies using conditional RNAi in *D. melanogaster *to analyze gene function in real time and a tissue-specific manner could overcome limitations of *in vitro* large-scale screening. In fact, the *in vivo *RNAi library for *Drosophila *flies has became commercially available [59], and a pilot genome-wide *in vivo *screen in *D. melanogaster *designed to identify genes involved in epithelial host defense against an intestinal bacterial pathogen was recently completed [38]. For the first time, this study showed that the JAK-STAT signaling pathway has an important role in host defense against infections with bacterial pathogens in the gut by regulating epithelial cell homeostasis.

## 7. Limitations of *Drosophila* Model of Fungal Infections

*Drosophila* offers unique advantages in dissecting immunopathogenesis of fungal diseases because of its powerful genetics and highly conserved immune pathways. Nonetheless, the fly model also has some obvious limitations. For example, implementing *Toll*-deficient flies as model for virulence testing in a Mycology laboratory requires some degree of training for proper maintenance and crossing of *Drosophila* stocks, and basic equipment for manipulating, anesthetizing, and infecting the animals. Alternatively, use of larger in size invertebrates, such as *Galleria mellonella*, which are easier to infect and allow for infection at mammalian temperatures, could overcome some technical difficulties of the *Drosophila* model [60]. However, because in *Galleria mellonella* genetic tools are not available and innate immune pathways are less well characterized, this model is not suitable for in-depth analysis of host-related factors mediating fungal pathogenesis.

**Figure 1:**
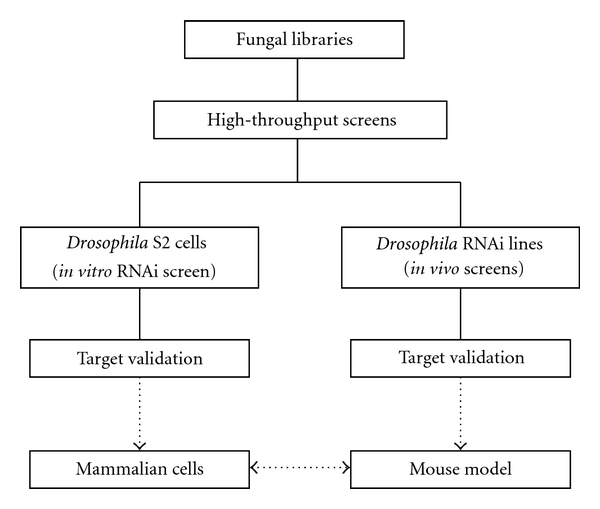
Prioritizing use of *Drosophila* model in selection of novel determinants of fungal pathogenicity in humans.

When compared to conventional animal models, the considerable difference in the anatomic structures of invertebrates and mammals raises questions on the pathophysiologic relevance of some *D. melanogaster *infection models. This may be particularly true for pathogens with life cycles adapted to mammalian hosts, or those that express their virulence mechanisms in a tissue-specific environment. For example, establishing a model of *Pneumocystis jirovecii *in invertebrate hosts [61] is not feasible. Nonetheless, even in mammalian hosts, some virulence attributes of pathogenicity may be dispensable to certain pathophysiologic settings or infection sites. For example, researchers recently showed that gliotoxin production was required for *A. fumigatus *pathogenicity in corticosteroid-immunosuppressed mice but not in neutropenic mice [62]. Furthermore, *D. melanogaster* lacks important constituents of human immunity, including a functional adaptive immune response, highly specialized innate immune cell subsets (e.g., dendritic cells, natural killer cells), and a complex network of cytokines, chemokines, and other effector molecules that have critical roles in orchestrating cell communication and regulation of inflammation and tolerance during infection. Overall, despite the considerable similarities in innate immune mechanisms, invertebrate models are not directly comparable with mammalian models. Thus, it is reasonable to speculate that some of the virulence attributes of *Aspergillus* and other filamentous fungi that affect mammals might not be important in invertebrate minihost models. Therefore, *Drosophila* must be viewed as a complementary, high-throughput genetic model, which could accelerate identification of novel host and pathogen determinants with a relevant role in development of fungal diseases in humans (Figure 1).

## 8. Future Directions in Fungal Immunology Research in *Drosophila*

The identification of the *Drosophila melanogaster Toll* signaling cascade and the subsequent characterization of mammalian *Toll*-like receptors (TLRs) have fundamentally altered our understanding of innate immunity. However, much remains to be learned on evolutionarily conserved antifungal immune defense mechanisms in *Drosophila* (Table 1). For example, whether immunostimulatory molecules of fungi other than b-glucans trigger immune recognition in *Drosophila* has not been elucidated. In addition, it is unknown whether and how *Drosophila* discriminates between pathogenic and opportunistic fungi. Similarly, the contribution of cellular immunity and *Toll*-independent mechanisms of antifungal host defense in fruit flies remains to be explored. Since the identification of mammalian TLRs, it became evident that nucleic acid sensing is an important aspect in pathogen recognition. Hence, dedicated endosomal TLRs and cytoplasmic pattern recognition receptors are specialized in sensing bacterial and viral nucleic acids and trigger robust inflammatory responses. Recent studies also demonstrate an important role for DNA neutrophil extracellular trap (NET) formation during bacterial and fungal infections [63]. In *Drosophila*, the role of nucleic acid sensing in immunity is largely unknown. Nonetheless, recent studies in other insects suggest that DNA NET formation is important for innate antibacterial immunity [64]. Finally, in humans, evolutionarily conserved antimicrobial peptides exert important immunomodulatory properties besides their direct effector function, by acting on various chemokine and signaling receptors [65, 66]. Therefore, whether *Drosophila *antimicrobial peptides retain a similar role is an important research direction in understanding the evolution of mammalian immune system.

## Figures and Tables

**Table 1:** Important research questions on antifungal immunity in *Drosophila*.

Which host defense mechanisms in *Drosophila *are important for fungal clearance before the induction of humoral immune responses? What is the contribution of cellular immunity in defense against fungi?	
How *Drosophila *immune system discriminates opportunistic from entomopathogenic fungi?	

Which fungal molecules other than b-glucans trigger activation of host immune responses in *Drosophila*?	

Are there any fungal virulence factors that exert immunosuppressive effects on *Toll* signaling or other immune signaling pathways? Is there any role of pathogen or self-nucleic acid sensing in *Drosophila* host defense?	

How *Drosophila* discriminates sensing of self- from non-self-immune activating molecules? Is there any evidence of the presence of endogenous ligands for *Drosophila *pattern recognition receptors?	

Are there any immune modulating properties of the antimicrobial peptides in *Drosophila? *	

Is there any role for antimicrobial peptide-DNA complex formation in insect immunity against fungi?	

Is there any cooperative activity between different *Drosophila* immune receptors?	
